# Stress response protein REDD1 promotes diabetes-induced retinal inflammation by sustaining canonical NF-κB signaling

**DOI:** 10.1016/j.jbc.2022.102638

**Published:** 2022-10-26

**Authors:** Siddharth Sunilkumar, Allyson L. Toro, Christopher M. McCurry, Ashley M. VanCleave, Shaunaci A. Stevens, William P. Miller, Scot R. Kimball, Michael D. Dennis

**Affiliations:** 1 Department of Cellular and Molecular Physiology, Penn State College of Medicine, Hershey, Pennsylvania, USA; 2 Department of Ophthalmology, Penn State College of Medicine, Hershey, Pennsylvania, USA

**Keywords:** diabetes, retina, DDIT4 (REDD1), inflammation, NF-kappa B, BCVA, best-corrected visual acuity, DR, diabetic retinopathy, HA, hemagglutinin, ICAM-1, intercellular adhesion molecule 1, IKK, IκB kinase, IP, immunoprecipitate, IκB, inhibitor of IκB, LPS, lipopolysaccharide, NF-κB, nuclear transcription factor κB, pUb, polyubiquitin, STZ, streptozotocin

## Abstract

Inflammation contributes to the progression of retinal pathology caused by diabetes. Here, we investigated a role for the stress response protein regulated in development and DNA damage response 1 (REDD1) in the development of retinal inflammation. Increased REDD1 expression was observed in the retina of mice after 16-weeks of streptozotocin (STZ)-induced diabetes, and REDD1 was essential for diabetes-induced pro-inflammatory cytokine expression. In human retinal MIO-M1 Müller cell cultures, REDD1 deletion prevented increased pro-inflammatory cytokine expression in response to hyperglycemic conditions. REDD1 deletion promoted nuclear factor erythroid-2-related factor 2 (Nrf2) hyperactivation; however, Nrf2 was not required for reduced inflammatory cytokine expression in REDD1-deficient cells. Rather, REDD1 enhanced inflammatory cytokine expression by promoting activation of nuclear transcription factor κB (NF-κB). In WT cells exposed to tumor necrosis factor α (TNFα), inflammatory cytokine expression was increased in coordination with activating transcription factor 4 (ATF4)-dependent REDD1 expression and sustained activation of NF-κB. In both Müller cell cultures exposed to TNFα and in the retina of STZ-diabetic mice, REDD1 deletion promoted inhibitor of κB (IκB) expression and reduced NF-κB DNA-binding activity. We found that REDD1 acted upstream of IκB by enhancing both K63-ubiquitination and auto-phosphorylation of IκB kinase complex. In contrast with STZ-diabetic REDD1^+/+^ mice, IκB kinase complex autophosphorylation and macrophage infiltration were not observed in the retina of STZ-diabetic REDD1^-/-^ mice. The findings provide new insight into how diabetes promotes retinal inflammation and support a model wherein REDD1 sustains activation of canonical NF-κB signaling.

Diabetic retinopathy (DR) is a significant ocular complication caused by diabetes that can progress to blindness. Retinal complications often develop upon loss of glucose homeostasis, with an estimated 103.12 million individuals suffering from DR and 18.83 million patients having vision threatening retinopathy (1). The pathogenesis of DR is complex and multi-factorial; however, it is well accepted that inflammation is a crucial factor in the progression of the retinal complications that are caused by diabetes (2). Inflammation is a protective immune response designed to facilitate tissue repair. Nevertheless, the chronic pro-inflammatory state of diabetes contributes to development and progression of DR. In diabetic patients without clinically visible signs of DR, expression of pro-inflammatory adhesion molecules and chemokines, such as intercellular adhesion molecule 1 (ICAM-1), C-C motif chemokine ligand (CCL2, also known as MCP-1), and CCL5 (also known as RANTES) are increased and contribute to leukostasis (3, 4, 5). This supports that the development of inflammation occurs early in disease progression. Indeed, clinical studies support the benefits of inhibiting specific pro-inflammatory molecules to address the development of neovascularization and macular edema in DR patients (6).

An abundance of literature has implicated the transcription factor nuclear factor κ-light-chain enhancer of activated B cells (NF-κB) as a key regulator of immune function and inflammatory responses (7). The NF-κB family of transcription factors [p65 (RelA), c-Rel, RelB, p50, and p52] form homodimers/heterodimers that determine the expression of an array of pro-inflammatory molecules. Canonical NF-κB activation involves phosphorylation of inhibitor of κB (IκB) by IκB kinase (IKK), which promotes IκB proteasomal degradation to allow nuclear translocation of the NF-κB RelA/p50 dimer. Noncanonical activation of RelB/p52 is mediated by IKK-dependent processing of p100 (8). NF-κB activation occurs in preclinical models of type 1 diabetes, and blocking its activity is beneficial in preventing DR pathology (9, 10, 11). Limited evidence also supports NF-κB activation in the retina of preclinical models of type 2 diabetes (12, 13). Although it was demonstrated nearly 2 decades ago that diabetes promotes NF-κB activation in the retina (14), the specific signaling events that lead to canonical or noncanonical NF-κB activation in DR have never been thoroughly resolved.

Diabetes promotes retinal expression of the stress response protein regulated in development and DNA damage 1 (REDD1) (11, 15, 16, 17). REDD1 expression is dominant in retinal Müller glia, where the protein contributes to a failed adaptive response of the retina to diabetes that includes gliosis, neurodegeneration, and the development of functional deficits in vision (15, 17). Indeed, therapeutic administration of an siRNA for REDD1 knockdown has demonstrated promise for improving best-corrected visual acuity (BCVA) in diabetic patients (18). REDD1 acts in the retina, at least in part, by preventing proper activation of the antioxidant transcription factor nuclear factor erythroid-2-related factor 2 (Nrf2) in response to diabetes (16). Beyond the retina, a number of recent studies have also demonstrated a role for REDD1 in the development of inflammation and NF-κB activation (19, 20, 21, 22, 23). More specifically, a recent study suggested that REDD1 mediated IKK-independent atypical NF-κB activation by sequestering IκB (22). Herein, we investigated a role for REDD1 in the development of diabetes-induced retinal inflammation and macrophage infiltration.

## Results

### Diabetes-induced REDD1 expression promotes markers of retinal inflammation

**Figure 1:**
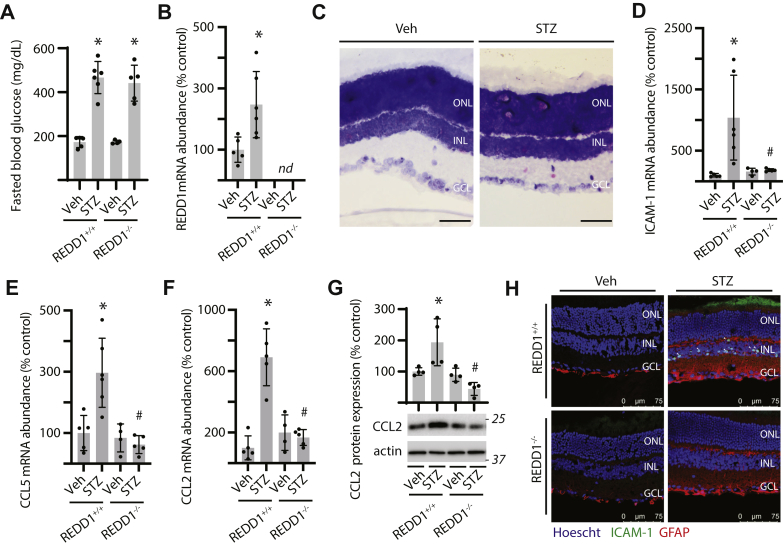
**REDD1 was required for increased inflammatory cytokine expression in the retina of diabetic mice.** Diabetes was induced in REDD1^+/+^ and REDD1^−/−^ mice by administration of streptozotocin (STZ). All analyses were performed 16 weeks after mice were administered STZ or a vehicle (Veh). *A*, fasting blood glucose concentrations were determined. *B*, REDD1 mRNA abundance in retinal tissue homogenate was determined by RT-PCR. C, REDD1 mRNA localization in the retina was examined by RNA-ISH (400*×* magnification; scale bar represents 50 μm). *D*–*F*, abundance of mRNAs encoding ICAM-1 (*D*), CCL5 (*E*), and CCL2 (*F*) was determined in retinal tissue homogenate by RT-PCR. *G*, CCL2 protein expression in retinal tissue homogenate was determined by Western blotting. Representative blots are shown. Molecular mass in kDa is indicated at *right* of each blot. *H*, ICAM-1 (*green*) and GFAP (*red*) were examined in retinal sections by immunofluorescence. Hoechst 33342 (*blue*) was used to visualize nuclei. Representative micrographs are shown (400*×* magnification; scale bar represents 75 μm). Values are presented as means ± SD. Data were analyzed in (*A*, *D*, and *E*–*G*) by two-way ANOVA, and pairwise comparisons were made using the Tukey's test for multiple comparisons. Data in (*B*) were analyzed by unpaired Student’s *t* test. ∗*p* < 0.05 *versus* Veh; #, *p* < 0.05 *versus* REDD1^+/+^; nd, not detected; CCL2, C-C motif chemokine ligand; GCL, ganglion cell layer; INL, inner nuclear layer; ICAM-1, intercellular adhesion molecule 1; ISH, *in situ* hybridization; ONL, outer nuclear layer.

After 16 weeks of diabetes, WT (REDD1^+/+^) and REDD1 knockout (REDD1^−/−^) mice exhibited similar increases in fasted blood glucose concentrations (Fig. 1*A*). REDD1 mRNA abundance was increased in retinal lysates from REDD1^+/+^ mice (Fig. 1*B*). Increased REDD1 mRNA expression in the retina of diabetic mice was visualized throughout retinal layers by *in situ* hybridization (Fig. 1*C*). The abundance of mRNA transcripts encoding ICAM-1 (Fig. 1*D*), CCL5 (Fig. 1*E*), and CCL2 (Fig. 1*F*) were also increased in the retina of diabetic mice in a manner that was REDD1-dependent. CCL2 protein expression was increased in the retina of diabetic REDD1^+/+^ mice, but not diabetic REDD1^−/−^ (Fig. 1*G*). Moreover, ICAM-1 protein expression in the inner retina and glial activation, as characterized by GFAP expression, were upregulated in diabetic REDD1^+/+^ mice (Figs. 1*H* and S1). Diabetes-induced expression of ICAM-1 and GFAP in the retina were attenuated in REDD1^−/−^ mice, as compared to REDD1^+/+^ mice.

### REDD1 deletion reduces inflammatory cytokine expression independently of Nrf2

**Figure 2:**
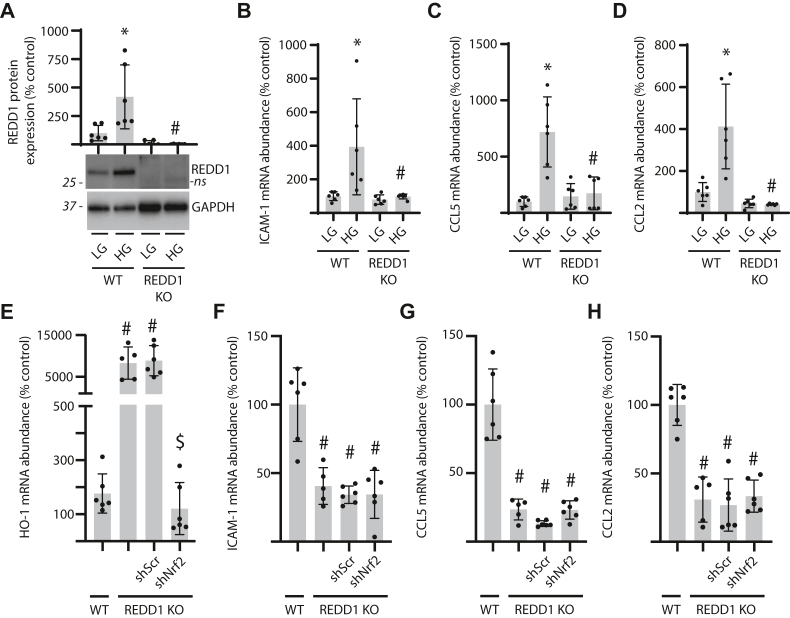
**Inflammatory cytokine expression was reduced in REDD1-deficient Müller glial cultures independently of Nrf2.***A*–*D*, WT and REDD1 KO human MIO-M1 cells were cultured in medium containing 5 mM glucose and exposed to medium containing either 30 mM glucose (HG) or 5 mM glucose and 25 mM mannitol as an osmotic control (LG) for 4 h. REDD1 protein expression in cell lysates was determined by Western blotting (*A*). Representative blots are shown. Molecular mass in kDa is indicated at *left* of each blot. ns, not specific. *E–H*, Nrf2 was knocked down in REDD1 KO MIO-M1 cells by stable expression of an shRNA (shNrf2). Control REDD1 KO cells expressed a scramble shRNA (shScr). All cells were exposed to HG for 4 h. Abundance of mRNAs encoding HO-1 (*E*), ICAM-1 (*B* and *F*), CCL5 (*C* and *G*), and CCL2 (*D* and *H*) was determined in cell lysates by RT-PCR. Values are presented as means ± SD. Data were analyzed by two-way ANOVA, and pairwise comparisons were made using the Tukey's test for multiple comparisons. ∗*p* < 0.05 *versus* LG; #, *p* < 0.05 *versus* WT; $, *p* < 0.05 *versus* shScr. CCL2, C-C motif chemokine ligand; ICAM-1, intercellular adhesion molecule 1; Nrf2, nuclear factor erythroid-2-related factor 2.

We recently demonstrated a role for REDD1 expression specifically in Müller glia in the development of retinal dysfunction in diabetic mice (17). To investigate a role for REDD1 in inflammatory cytokine production by human cells, MIO-M1 Müller glia were exposed to hyperglycemic culture conditions. Hyperglycemic conditions promoted REDD1 protein expression (Fig. 2*A*) and increased the abundance of mRNAs encoding ICAM-1 (Fig. 2*B*), CCL5 (Fig. 2*C*), and CCL2 (Fig. 2*D*). Increased inflammatory cytokine expression in cells exposed to hyperglycemic conditions required REDD1. Inflammation has long been associated with oxidative stress and we recently demonstrated protective effects of REDD1 deletion that were mediated by enhanced Nrf2 signaling (16). In REDD1-deficient cells exposed to hyperglycemic conditions, Nrf2 knockdown reduced expression of the Nrf2 target gene Heme Oxygenase-1 (HO-1) (Fig. 2*E*). However, Nrf2 knockdown did not impact the abundance of mRNAs encoding ICAM-1 (Fig. 2*F*), CCL5 (Fig. 2*G*), or CCL2 (Fig. 2*H*) in REDD1-deficient cells exposed to hyperglycemic conditions.

### REDD1 sustains TNFα-induced NF-κB signaling

**Figure 3:**
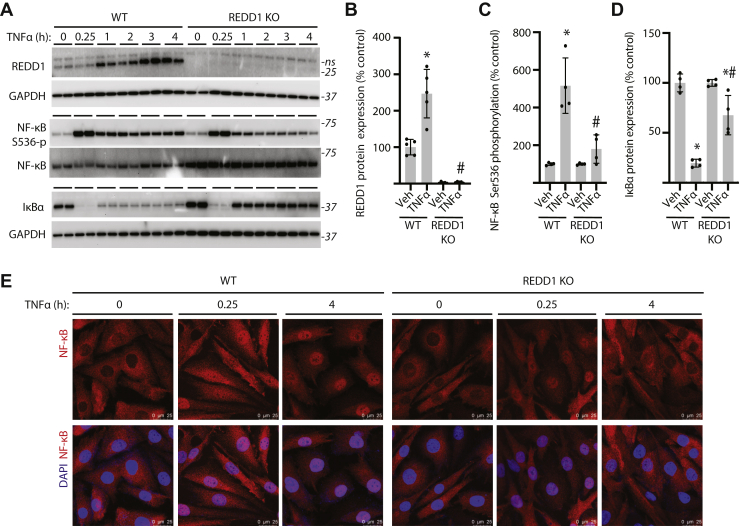
**REDD1 sustained NF-κB signaling in Müller glial cultures exposed to TNF⍺.** WT and REDD1 KO human MIO-M1 cells were exposed to medium supplemented with TNF⍺ for up to 4 h. *A*, REDD1 expression, NF-κB phosphorylation at S536, and IκB⍺ expression were determined in cell lysates by Western blotting. Representative blots are shown. Molecular mass in kDa is indicated at *right* of each blot. REDD1 expression (*B*), NF-κB phosphorylation (*C*), and IκB⍺ expression (*D*) were quantified after 4 h of exposure to TNF⍺ or vehicle (Veh). Values are presented as means ± SD. Data were analyzed by two-way ANOVA, and pairwise comparisons were made using the Tukey's test for multiple comparisons. ∗*p* < 0.05 *versus* Veh; #, *p* < 0.05 *versus* WT. *E*, nuclear localization of NF-κB p65 was determined by immunofluorescence. Nuclei were visualized with DAPI (600*×* magnification; scale bar represents 25 μm). IκB, inhibitor of IκB; ns, not specific; NF-κB, nuclear transcription factor κB.

To explore the impact of REDD1 on NF-κB signaling, WT and REDD1 KO MIO-M1 cells were exposed to TNFα. In WT cells, REDD1 expression was increased after 1 to 4 h of exposure to TNFα (Fig. 3, *A* and *B*). Acute TNFα exposure (*i.e.*, 0.25 h) promoted rapid phosphorylation of NF-κB at S536 and reduced expression of IκBα in both WT and REDD1 KO cells (Fig. 3*A*). However, after 4 h of TNFα exposure, phosphorylation of NF-κB (Fig. 3, *A* and *C*) and reduced Iκ-Bα expression (Fig. 3, *A* and *D*) were sustained in WT, but not in REDD1 KO cells. In both WT and REDD1 KO cells, TNFα caused rapid localization of NF-κB to the nucleus (Fig. 3*E*). With more prolonged exposure to TNFα, NF-κB remained localized to the nucleus in WT cells, whereas the transcription factor returned to the cytoplasm in REDD1 KO cells. The data support that REDD1 is required for sustained activation of NF-κB.

### IKK activity is required for REDD1-dependent NF-κB activation and inflammatory cytokine expression

**Figure 4:**
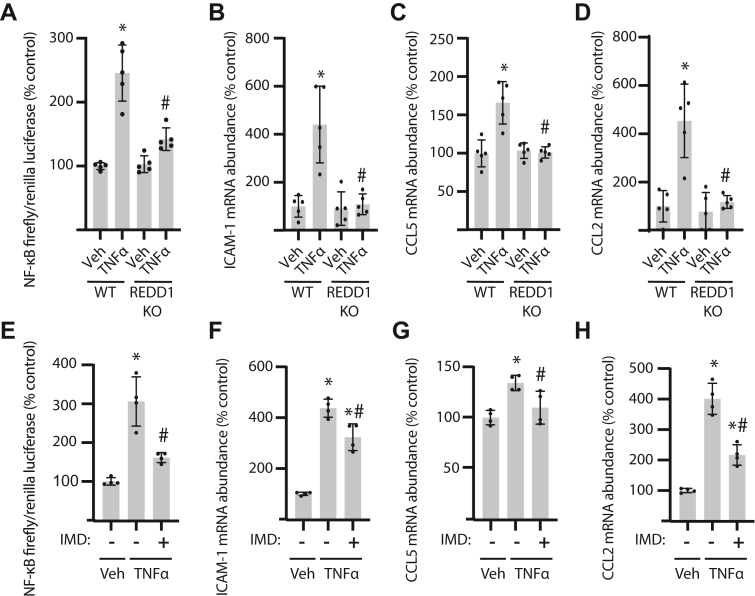
**REDD1-dependent NF-κB activation and inflammatory cytokine expression required IKK.***A*–*H*, WT and REDD1 KO human MIO-M1 cells were exposed to medium supplemented with TNF⍺ or vehicle (Veh) for 4 h. *E*–*H*, WT MIO-M1 cells were exposed to the IKKβ inhibitor IMD0354 (IMD) for 1 h prior to the addition of TNF⍺. NF-κB activity was measured in lysates from cells expressing NF-κB firefly luciferase/*Renilla* luciferase reporter plasmids by dual luciferase assay (*A* and *E*.). Abundance of mRNAs encoding ICAM-1 (*B* and *F*), CCL5 (*C* and *G*), and CCL2 (*D* and *H*) was determined in cell lysates by RT-PCR. Values are presented as means ± SD. Data were analyzed by either two-way (*A–D*) or one way (*E–H*) ANOVA, and pairwise comparisons were made using the Tukey's test for multiple comparisons. ∗*p* < 0.05 *versus* Veh; #, *p* < 0.05 *versus* WT or TNF⍺ alone. CCL2, C-C motif chemokine ligand; IKK, IκB kinase; ICAM-1, intercellular adhesion molecule 1; NF-κB, nuclear transcription factor κB.

To further examine NF-κB activity in Müller glia, a NF-κB luciferase reporter system was employed. TNFα promoted NF-κB reporter activity in WT cells, whereas REDD1 deletion prevented the effect (Fig. 4*A*). TNFα also enhanced the abundance of mRNAs encoding ICAM-1 (Fig. 4*B*), CCL5 (Fig. 4*C*), and CCL2 (Fig. 4*D*) in WT cells. Remarkably, REDD1 deletion prevented an increase in inflammatory cytokine expression in cells exposed to TNFα. Similarly, pharmacological IKKβ inhibition suppressed TNFα-induced NF-κB reporter activity in WT cells (Fig. 4*E*) and reduced expression of ICAM-1 (Fig. 4*F*), CCL5 (Fig. 4*G*), and CCL2 (Fig. 4*H*).

### REDD1 promotes canonical NF-κB signaling

**Figure 5:**
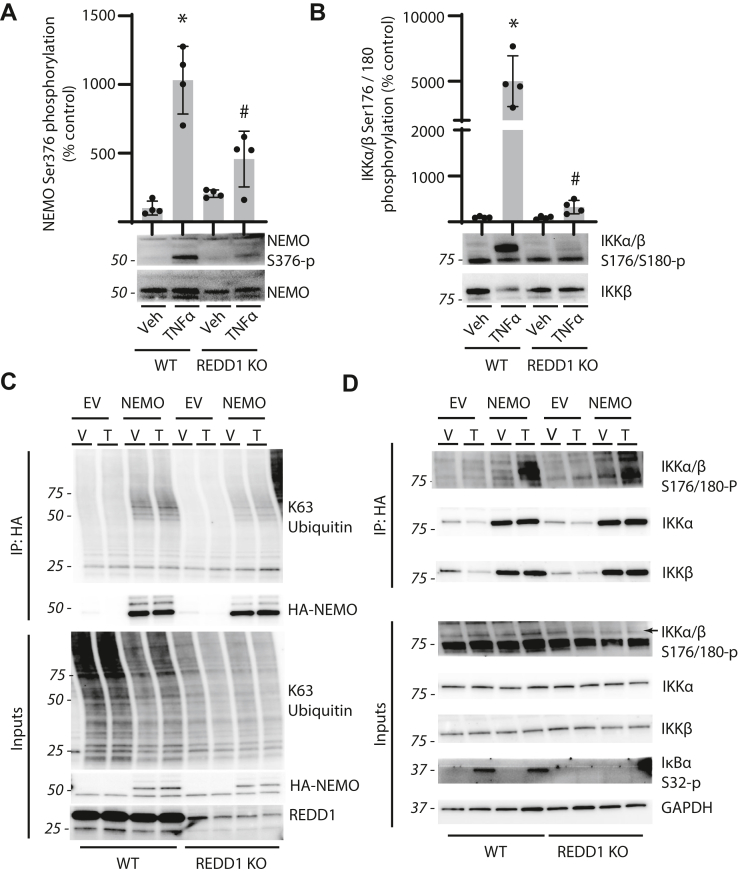
**REDD1 promoted canonical NF-κB signaling.***A* and *B*, WT and REDD1 KO MIO-M1 cells were exposed to medium supplemented with TNFα or vehicle (Veh) for 4 h. Phosphorylation of NEMO at S376 (*A*) and IKKα/β at S176/180 (*B*) was determined by Western blotting. Values are presented as means ± SD. Data were analyzed by two-way ANOVA, and pairwise comparisons were made using the Tukey's test for multiple comparisons. ∗*p* < 0.05 *versus* Veh; #, *p* < 0.05 *versus* WT. *C* and *D*, HA-tagged NEMO (HA-NEMO) or an empty vector control (EV) was expressed in HEK293 cells by transient transfection. Cells were exposed to medium supplemented with TNFα or Veh for 1 h. HA-NEMO was immunoprecipitated from cell lysates by affinity purification. Cell lysate (5% of input) and HA-tag immunoprecipitate (IP: HA) was examined by Western blotting. Representative blots are shown. Molecular mass in kDa is indicated at *left* of each blot. HA, hemagglutinin; IKK, IκB kinase; NF-κB, nuclear transcription factor κB; TNFα, tumor necrosis factor α.

Canonical NF-κB signaling involves activation of the IKK complex that consists of two kinases (IKKα and IKKβ) and a regulatory subunit (NEMO/IKKγ). TNFα exposure enhanced phosphorylation of both NEMO at S376 (Fig. 5*A*) and IKKα/β at S176/S180 (Fig. 5*B*) in WT MIO-M1 cells, but not in REDD1 KO cells. K63-linked nondegradable polyubiquitin (pUb) chains regulate canonical NF-κB signaling (24, 25), with direct ubiquitination of NEMO positively modulating IKK complex activation (26, 27, 28). To determine the impact of REDD1 on K63-specific ubiquitination of NEMO, hemagglutinin (HA)-tagged NEMO was expressed in HEK239 cells. K63-pUb was detected in the HA-NEMO immunoprecipitate (IP) from cell lysates (Fig. 5*C*). As compared to WT cells, K63-pUb was reduced in the HA-NEMO IP from REDD1 KO cells. Surprisingly, IKKα and IKKβ coimmunoprecipitation with HA-NEMO was unaffected by REDD1 deletion or TNFα exposure (Fig. 5*D*). However, enhanced phosphorylation of IKKα/β was observed in the HA-NEMO IP from WT cells after TNFα exposure, whereas a similar effect was not observed in REDD1 KO cells. Further, phosphorylation of IKKα/β (S176/S180) and IκB at S32 were enhanced upon TNFα exposure in a manner that was dependent on REDD1 expression.

### REDD1 deletion suppressed canonical NF-κB signaling in the retina of diabetic mice

**Figure 6:**
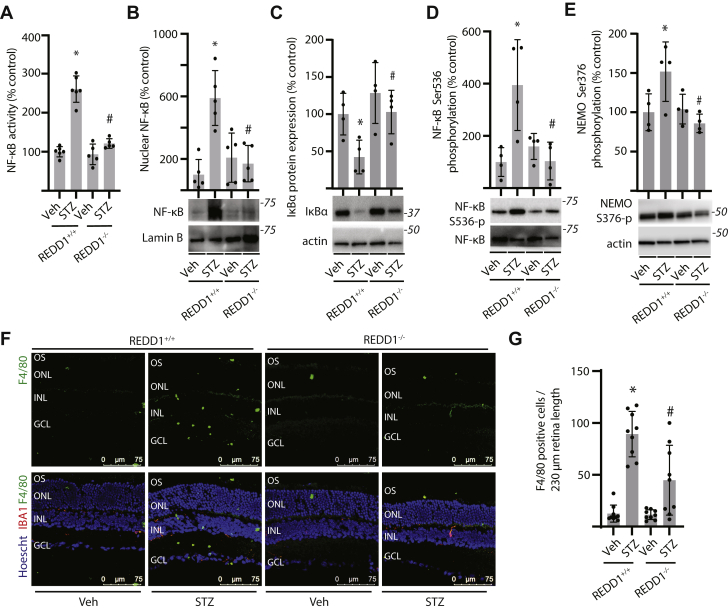
**REDD1 deletion prevented NF-κB activation and macrophage infiltration in the retina of diabetic mice.** Diabetes was induced in REDD1^+/+^ and REDD1^−/−^ mice by administration of streptozotocin (STZ) or a vehicle (Veh) control. All analyses were performed after 16 weeks of diabetes. *A*, NF-κB activity was determined in nuclear isolates obtained from retinal lysates by DNA-binding ELISA. *B*, NF-κB p65 protein expression in nuclear isolates was determined by Western blotting. Representative blots are shown. Molecular mass in kDa is indicated at *right* of each blot. *C–E*, IκBα expression (*C*), NF-κB phosphorylation at S536 (*D*), and NEMO phosphorylation at S376 (*E*) were evaluated in retinal tissue homogenate by Western blotting. *F*, F4/80 (*green*) and IBA1 (*red*) were examined in retinal sections by immunofluorescence. Hoechst 33342 (*blue*) was used to visualize nuclei. Representative micrographs are shown (400*×* magnification; scale bar represents 75 μm). *G*, F4/80-positive cells in (*F*) were counted. Values are presented as means ± SD. Data were analyzed by two-way ANOVA, and pairwise comparisons were made using the Tukey's test for multiple comparisons. ∗*p* < 0.05 *versus* Veh; #, *p* < 0.05 *versus* REDD1^+/+^; GCL, ganglion cell layer; IκB, inhibitor of IκB; INL, inner nuclear layer; NF-κB, nuclear transcription factor κB; ONL, outer nuclear layer; OS, photoreceptor outer segments.

To investigate a role for REDD1 in diabetes-induced NF-κB activation, nuclear isolates from whole retina were examined using an ELISA that measures the binding of NF-κB p65 to an oligonucleotide encoding the κB consensus motif. Diabetes promoted NF-κB activity (Fig. 6*A*) and increased nuclear NF-κB p65 expression (Fig. 6*B*) in the retina of REDD1^+/+^ mice, but not in REDD1^−/−^ mice. IκB expression was also reduced in the retina of diabetic REDD1^+/+^ mice, as compared to nondiabetic REDD1^+/+^ mice (Fig. 6*C*). As compared to diabetic REDD1^+/+^ mice, IκB expression was increased in the retina of diabetic REDD1^−/−^ mice. Diabetes also promoted phosphorylation of NF-κB at S536 (Fig. 6*D*) and NEMO at S376 (Fig. 6*E*), which were also dependent on REDD1. To correlate changes in NF-κB with retinal inflammation, leukostasis was examined in retinal sections. In the retina of REDD1^+/+^ mice, diabetes resulted in macrophage infiltration into the inner retina (Fig. 6, *F* and *G*). However, as compared to nondiabetic REDD1^−/−^ mice, macrophage infiltration was not altered in the retina of diabetic REDD1^−/−^ mice.

### Activating transcription factor 4 is necessary for TNFα-induced REDD1 expression

**Figure 7:**
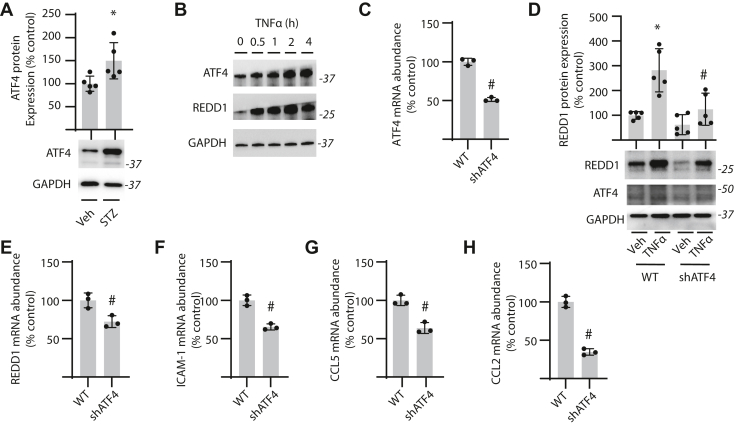
**ATF4 was necessary for TNFα-induced REDD1 expression.***A*, diabetes was induced in mice by administration of streptozotocin (STZ) or a vehicle (Veh) control. After 16 weeks of diabetes, ATF4 protein expression was examined in retinal lysates by Western blotting. Representative blots are shown. Molecular mass in kDa is indicated at *right* of each blot. *B*, MIO-M1 cells were exposed to medium supplemented with TNFα or vehicle (Veh) for up to 4 h. ATF4 and REDD1 protein expression were examined by Western blotting. *C*, ATF4 mRNA was examined in MIO-M1 cells expressing an shRNA targeting the ATF4 mRNA (shATF4) *versus* WT control cells by RT-PCR. *D*, WT and shATF4 cells were exposed to TNFα for 2 h and REDD1 protein expression was determined by Western blotting. *E–H*, REDD1 (*E*), ICAM-1 (*F*), CCL5 (*G*), and CCL2 (*H*) mRNA abundances were determined in WT and shATF4 cells by RT-PCR. Values are presented as means ± SD. Data in (*A*, *C*, and *E*–*H*) were analyzed by unpaired Student’s *t* test. Data in (*D*) were analyzed by two-way ANOVA, and pairwise comparisons were made using the Tukey's test for multiple comparisons. ∗*p* < 0.05 *versus* Veh; #, *p* < 0.05 *versus* WT. ATF4, activating transcription factor 4; CCL2, C-C motif chemokine ligand; ICAM-1, intercellular adhesion molecule 1; TNFα, tumor necrosis factor α.

To investigate potential upstream mediators that promote REDD1 mRNA expression in the retina, a role for the transcription factor activating transcription factor 4 (ATF4) was examined. ATF4 is a well-documented regulator of stress-induced REDD1 mRNA transcription (29, 30, 31). ATF4 protein expression was increased in retinal lysates from streptozotocin (STZ)-diabetic mice as compared to their littermate controls (Fig. 7*A*). When MIO-M1 cells were exposed to TNFα, ATF4 and REDD1 protein expression coordinately increased (Fig. 7*B*). In cells expressing an shRNA targeting the ATF4 mRNA, ATF4 expression was suppressed (Fig. 7*C*), and the stimulatory effect of TNFα on REDD1 expression was absent (Fig. 7*D*). In addition to reducing REDD1 mRNA abundance (Fig. 7*E*), ATF4 knockdown also suppressed the abundance of mRNAs encoding ICAM-1 (Fig. 7*E*), CCL5 (Fig. 7*F*), and CCL2 (Fig. 7*G*). Together, the data support ATF4-dependent REDD1 expression in the regulation of inflammatory cytokine production.

## Discussion

**Figure 8:**
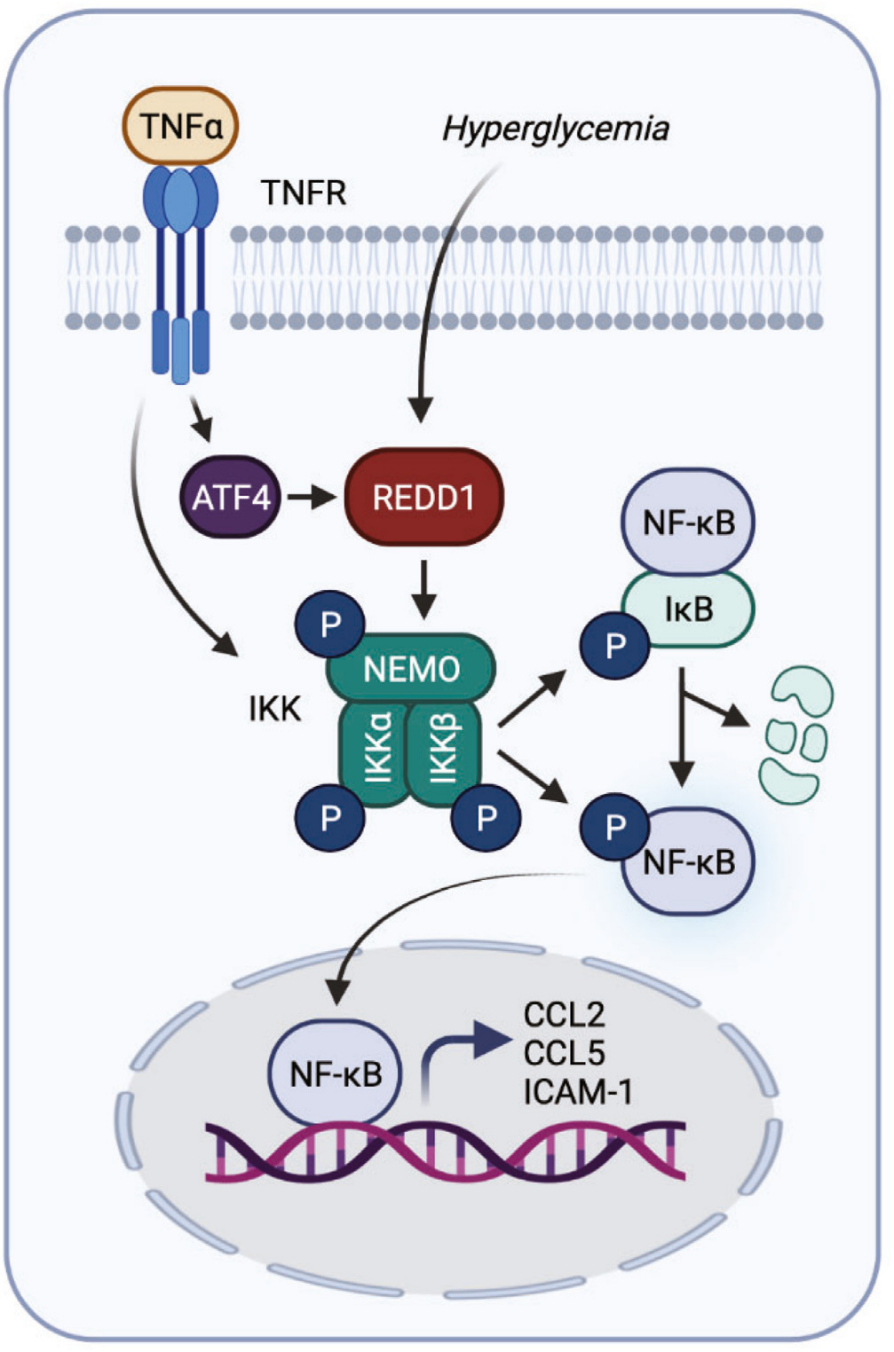
**REDD1 sustains NF-κB signaling to promote retinal inflammation.** Working model for the role of REDD1 in diabetes-induced activation of NF-κB signaling and enhanced inflammatory cytokine production by Müller glia. Graphic created with BioRender.com. NF-κB, nuclear transcription factor κB.

Herein, we investigated a role for REDD1 in the development of diabetes-induced retinal inflammation. Increased REDD1 expression was observed in the retina of mice after 16-weeks of STZ-diabetes, as well as in human Müller glia cultures exposed to hyperglycemic culture conditions. Diabetes promoted NF-κB signaling, enhanced pro-inflammatory cytokine expression, and lead to the development of retinal leukostasis. However, REDD1 deletion reduced IKK activation, suppressed nuclear localization of NF-κB, and attenuated both inflammatory cytokine expression and macrophage infiltration of the inner retina in diabetic mice. Overall, the studies provide evidence that REDD1 acts to promote retinal inflammation by sustaining activation of canonical NF-κB signaling (Fig. 8).

DR is diagnosed by clinically visible microvascular abnormalities in the retina and thus has principally been investigated as a microvascular complication. However, evidence supports that gliosis, neuroinflammation, neurodegeneration, and loss of neurovascular coupling are critical elements of disease pathogenesis (32). In fact, neuroglial deficits can precede and even predict the visible signs of microvascular disease in diabetic patients (33, 34). Specificity of REDD1 expression in human retina is consistent with markers of Muller glia, and conditional deletion of REDD1 in Muller glia of mice prevents the increase in retinal REDD1 expression with STZ-diabetes (17). Enhanced REDD1 mRNA was observed throughout the retinal layers after 16 weeks of STZ-diabetes. Muller glia extend radially across the entire retina, thus the localization of REDD1 mRNA in retinal sections was not unexpected. Retinal Müller glia play a central role in the development of inflammation in DR (2). Müller glia provide critical homeostatic and trophic support to maintain both activity of retinal neurons and integrity of the blood-retinal barrier. In response to diabetes, Müller cells become activated and secrete a range of pro-inflammatory factors that are NF-κB target genes (35). Herein, expression of ICAM-1, CCL5, and CCL2 were increased in both the retina of STZ-diabetic mice and in retinal Müller glia cultures exposed to TNF⍺ in coordination with activation of NF-κB.

NF-κB serves as a key mediator of the retinal inflammatory response to diabetes (36, 37, 38). It has long been established that diabetes promotes retinal NF-κB activation in both diabetic patients (14) and in preclinical models (39, 40). However, the underlying mechanisms responsible for enhanced nuclear localization of NF-κB in the context of DR are not well delineated. Only recently was enhanced NF-κB phosphorylation reported in the retina of Ins2^Akita^ mice (41) and STZ-diabetic rats (42) and mice (43). Limited reports of enhanced IκB phosphorylation and reduced IκB expression in the retina of STZ-diabetic rodents support activation of canonical NF-κB signaling (43, 44). Canonical NF-κB signaling depends on activation of the IKK complex, which includes two kinase subunits (IKKα/β) and the regulatory subunit NEMO. In particular, IKKβ and NEMO mediate canonical NF-κB signaling, whereas noncanonical signaling is dependent on IKKα. Increased IKKβ phosphorylation was recently reported in retinal lysates from STZ-diabetic rats (45). Herein, increased NF-κB DNA-binding activity was observed in the retina of STZ-diabetic mice in coordination with nuclear localization and phosphorylation of NF-κB p65. Moreover, diabetes attenuated retinal IκB expression and enhanced autophosphorylation of NEMO within the IKK complex.

A growing body of literature supports that the stress response protein REDD1 plays a critical role in the development of diabetes-induced retinal defects. Indeed, REDD1 deletion prevents retinal pathology and functional deficits in vision in STZ-diabetic mice (11, 15, 16). It is well established that REDD1 mediates the cellular stress response to a number of adverse conditions, including hyperglycemia (46, 47). In both the retina of diabetic mice and in human retinal cell cultures exposed to hyperglycemic conditions, REDD1 expression was upregulated in association with pro-inflammatory signaling. Deletion of REDD1 prevented a pro-inflammatory response to diabetes and hyperglycemic conditions, supporting a role for REDD1 in development of inflammation. This is consistent with prior studies on endotoxemia pathophysiology that demonstrate an essential role for REDD1 in lipopolysaccharide (LPS)-induced inflammation (21, 22).

Oxidative stress resulting from an imbalance between the production of reactive oxygen species and their elimination by antioxidants contributes to the development of retinal inflammation with diabetes (39). Upregulation of REDD1 has been linked to multiple disease models that involve the development of oxidative stress, including DR (47). Retinal cells combat the development of oxidative stress through activation of the Nrf2 antioxidant response. Nrf2 deletion promotes the development of inflammation with ischemia-reperfusion injury (48). Moreover, knockdown of Nrf2 and its downstream targets HO-1 and NQO1 increases NF-κB–mediated inflammation in monocytes exposed LPS (49). REDD1 acts to negatively regulate Nrf2 activity in the retina of diabetic mice by promoting nuclear exclusion and degradation of the transcription factor (16). As compared to WT Müller cells exposed to hyperglycemic conditions, REDD1 deletion promoted Nrf2 hyperactivation in coordination with blunted expression of ICAM-1, CCL5, and CCL2. Nrf2 knockdown in REDD1-deficient cells restored HO-1 expression to a level that was similar to that observed in WT cells. However, Nrf2 knockdown did not enhance expression of ICAM-1, CCL5, and CCL2 in REDD1-deficient cells exposed to hyperglycemic conditions. This supports that the reduced inflammatory cytokine expression with REDD1 ablation was independent of Nrf2 hyperactivation.

A role for REDD1 in NF-κB–mediated inflammation has been noted in mice exposed to LPS or cigarette smoke (19, 21, 22). Lee *et al.* (2018) demonstrated that REDD1 promotes atypical NF-κB activation by IκBα sequestration (22). In macrophages exposed to LPS for 24 h, REDD1 interferes with IκBα binding to NF-κB to promote nuclear translocation and activity of the transcription factor (22). Prior work suggests that LPS activates NF-κB *via* both an early IKK-dependent mechanism, as well as through delayed IKK-independent mechanism (50). Notably, Lee *et al.* observed a suppressive effect of REDD1 on NF-κB p65 phosphorylation in macrophages exposed to LPS for only 30 min, despite absence of IκBα expression at that timepoint (22). The observation suggests the possibility that REDD1 may also act independently of IκBα sequestration. The studies herein extend on the prior work by demonstrating that REDD1 sustained canonical NF-κB signaling by enhancing IKK activation to suppress IκBα expression. REDD1 deletion reduced both auto-phosphorylation and K63-ubiquitination of the IKK complex. Together with the prior report (22), the findings support a model wherein REDD1 acts by both enhancing IKK-dependent degradation of IκBα to promote NF-κB nuclear translocation and through IKK-independent sequestration of newly synthesized IκBα. In addition to promoting the transcription of pro-inflammatory cytokines, NF-κB also mediates transcription of IκBα, which facilitates feedback inhibition of the signaling pathway (51). In REDD1-deficient Müller glia cultures, TNFα-induced NF-κB phosphorylation and the reduction in IκBα expression were resolved more quickly than in WT cells. This suggests that REDD1 uncouples autoregulation of NF-κB activation and provides an additional molecular checkpoint for the termination of pro-inflammatory signaling.

We recently provided evidence that increased retinal REDD1 protein expression results from impaired lysosomal proteolysis of REDD1 protein as a consequence of redox stabilization (52). After 6 weeks of STZ-diabetes, enhanced REDD1 protein expression in retinal lysates is observed in the absence of a change in REDD1 mRNA expression (52). By contrast, 16 weeks of STZ-diabetes resulted in upregulated REDD1 mRNA abundance. The observation suggests that with prolonged diabetes duration, redox stabilization of REDD1 is potentially exacerbated by a transcriptional effect. Prior reports demonstrate that retinal REDD1 expression is transcriptionally upregulated in response to hypoxic stress in a murine model of retinopathy of prematurity (53). Moreover, evidence supports that REDD1 is a transcriptional target of ATF4 (29), HIF-1α (54), p53 (55), and even RelB (56). Herein, increased retinal REDD1 mRNA abundance was observed coincident with enhanced ATF4 protein expression. ATF4 knockdown prevented an increase in REDD1 expression in Müller cells exposed to TNFα and attenuated inflammatory cytokine expression. The data support a prior study that identified an essential role for ATF4 in diabetes-induced inflammatory cytokine production by Müller glial (57). More specifically, ATF4 expression localizes to Müller cells in the retina of STZ-diabetic mice, and expression of a dominant negative ATF4 variant reduces inflammatory cytokine expression in both the retina of diabetic mice and in Müller cells exposed to hyperglycemic conditions (57). The studies here extend on the prior work by demonstrating a key role for REDD1 and NF-κB acting downstream of ATF4 to promote Müller glial expression of pro-inflammatory cytokines.

Overall, the findings here provide new insight into the molecular mechanisms whereby diabetes contributes to the development of retinal inflammation. In the retina of diabetic mice, REDD1 expression was increased in coordination with NF-κB activation, pro-inflammatory cytokine expression, and macrophage infiltration. REDD1 deletion reduced NF-κB activation in the retina of diabetic mice and prevented the development of leukostasis. The data support that therapeutic intervention to suppress REDD1 expression may improve retinal pathology in diabetic patients. Therapeutically, DR is principally addressed by blockade of vascular endothelial growth factor (58). A major limitation of these interventions is that vascular endothelial growth factor suppression largely addresses the microvascular dysfunction and neovascularization that characterize later stages of disease progression. The studies here support that REDD1 suppression may represent a therapeutic strategy to prevent the development of retinal inflammation. Indeed, administration of an intravitreally administered siRNA targeting the REDD1 mRNA (PF-04523655) in patients with diabetic macular edema showed a trend toward improvement in BCVA when compared to focal/grid laser (+5.8 letters with 3 mg PF-04523655 *versus* +2.4 letters with laser, *p* = 0.08) (18). In a secondary analysis of patients that completed a 12-months follow up, mean improvement in BCVA with 3 mg PF-04523655 was superior to laser intervention. The proof-of-concept studies here are consistent with a mechanism of action wherein REDD1 suppression improves visual acuity in diabetic patient by preventing sustained NF-κB activation and reducing the retinal inflammatory response to diabetes.

## Experimental procedures

### Animals

Male WT (REDD1^+/+^) and REDD1 KO (REDD1^−/−^) B6;129 mice (53) were maintained on a 12:12-h reverse light dark cycle. Diabetes was induced at 6 weeks of age by administering 50 mg/kg STZ intraperitoneally for five consecutive days. Littermate control mice were injected with equivalent volumes of sodium citrate buffer. Two weeks after injections, diabetic phenotype was confirmed by fasting blood glucose concentrations >250 mg/dL. At 16 weeks of diabetes, mice were euthanized, and retina and whole eyes were extracted. All procedures were approved by the Penn State College of Medicine Institutional Animal Care and Use Committee and were in accordance with the ARVO statement on the ethical use of animals in ophthalmological research.

### Cell culture

MIO-M1 human Müller cells were obtained from the UCL Institute of Ophthalmology. MIO-M1 cells deficient for REDD1 (REDD1 KO) were generated using CRISPR/Cas9 genome editing as previously described (59, 60). MIO-M1 cultures were maintained in Dulbecco’s modified Eagle’s medium (DMEM, Thermo Fisher Scientific) containing 5.6 mM glucose and supplemented with 10% heat inactivated fetal bovine serum and 1% penicillin-streptomycin. MIO-M1 cells stably expressing an shRNA targeting Nrf2 (5′-CCGGG-CTCCTACTGTGATGTGAAATCTCGAGATTTCACATCACAGTAGGAGCTTTTT-3′) or ATF4 (5′-CCGGGCCTAGGTCTCTTAGATGATTCTCGAGAATCATCTAAGAGACCTAGGCTTTTT-3′) were generated as previously described (16). Cells expressing pLKO.1-TRC [provided by David Root (Addgene Plasmid #10879)] were used as an shRNA control. To model hyperglycemia, culture medium was supplemented with either 24.4 mM glucose or 24.4 mM mannitol as an osmotic control. Cells were transfected using Lipofectamine 2000 (Life Technologies). Plasmids included pCMV5 vector, pCMV-HA-REDD1, pRL-Renilla luciferase (Promega). NF-κB-TATA luciferase reporter and pCMV4-HA-NEMO (IKKγ) plasmids were kindly provided by Dr Edward Harhaj (Penn State College of Medicine). Where indicated, cell culture medium was supplemented with recombinant human TNFα (20 ng/ml; Sigma) or 1 μM N-(3,5-Bis-trifluoromethylphenyl)-5-chloro-2-hydroxybenzamide (IMD0354; Cayman chemicals).

### Immunofluorescent microscopy

Whole eyes were excised, and corneas were punctured, followed by incubation in 4% paraformaldehyde (pH 7.5) for 30 min. Eyes were washed with PBS and incubated at 4 °C in 30% sucrose solution containing 0.05% sodium azide. Eyes were embedded in optimal cutting temperature compound, flash frozen, and sectioned. Cryosections (10 μm) were fixed in 2% paraformaldehyde, then permeabilized in PBS with 0.1% Triton-X-100, and blocked in 10% normal donkey serum. Sections were labeled with the appropriate antibodies (Table S1). All sections were counter stained with 1.6 μmol/L Hoechst. Slides were mounted with Fluoromount aqueous mounting media (Sigma-Aldrich) and imaged with a confocal laser microscope (Leica SP8; Leica) using frame-stack sequential scanning. As a control for nonspecific secondary antibody binding, primary antibody was omitted in analysis of some retinal sections (Fig. S2). ImageJ was used to obtain macrophage counts after thresholds were set to isolate positive cells and single stained population counts were then confirmed manually.

### In Situ hybridization

REDD1 mRNA was visualized using the RNAscope 2.5 HD Assay-RED detection kit and the RNAscope Probe-Mm-Ddit4 probe (Advanced Cellular Diagnostics) targeting nucleotides 133-1480 of NM_029083.2. RNA-*in situ* hybridization was carried out on 10 μm whole eye cryosections following the manufacturer's protocol. REDD1 mRNA hybridization was carried out on protease and heat-treated sections. Sequential hybridization with preamplifier, amplifier, and alkaline phosphatase–labeled oligos was carried out followed by the application of a chromogenic substrate. Tissue was counter-stained with Mayer’s Hematoxylin and 0.02% ammonia water, and micrographs were captured using an AmScope T720Q brightfield microscope.

### Nuclear fractionation

Retinas were homogenized in ice-cold hypotonic buffer (20 mM Hepes pH 7.5, 5 mM NaF, 10 μM sodium molybdate, 0.1 mM EDTA, 0.5% Nonidet P-40, and 1% protease-phosphatase inhibitors). Homogenates were centrifuged at 228*g* for 5 min at 4 °C to pellet nuclei. The nuclear pellet was resuspended in 50 μL complete lysis buffer (Active Motif) and incubated at 4 °C for 30 min on a shaking platform. Lysates were centrifuged at 14,000*g* for 10 min at 4 °C. Nuclear extract was collected as the supernatant fraction.

### DNA-binding ELISA

NF-κB activity was quantified using a colorimetric NF-κB p65 DNA-binding ELISA (Trans AM NF-κB p65; Active Motif). Briefly, 20 μg of nuclear protein was incubated for 1 h in the presence of an immobilized oligonucleotide encoding the NF-κB consensus sequence. Binding of the p65 subunit was quantified using an anti-p65 primary antibody and horseradish peroxidase–conjugated secondary. The absorbance (λmax 450 nm) was recorded using Spectra Max M5 plate reader (Molecular Devices).

### Immunoprecipitations

Immunoprecipitations were performed on 1000*g* supernatant fractions of cell lysate. HA-tag immunoprecipitation was performed using EZview Red Anti-HA affinity gel (Sigma). Beads were washed with lysis buffer [1 mM EDTA, 5 mM EGTA, 10 mM MgCl_2_, 50 mM β-glycerophosphate, and 0.1% NP-40] and blocked with lysis buffer containing 1% bovine serum albumin. Cells were harvested in lysis buffer supplemented with 2 mM N-ethylmaleimide, 10 mM sodium pyrophosphate, 1 mM benzamidine, 200 mM sodium vanadate, and protease inhibitor mixture (10 μL/mL) and lysed for 30 min at 4 °C. Cell supernatants were collected by centrifuging lysates for 3 min at 1000*g* and incubated with the appropriate affinity resin overnight at 4 °C. Affinity resins were washed with cold lysis buffer, resuspended in 1× SDS sample buffer, and boiled for 5 min. The IP was subjected to Western blot analysis.

### Western blotting

Retinas were flash frozen in liquid nitrogen and homogenized as previously described (11). Retinal protein was quantified by DC protein assay. Equal protein from cell lysates, retinal homogenates, or nuclear extracts were combined with Laemmli buffer, boiled, and fractionated in Criterion Precast 4 to 20% gels (Bio-Rad Laboratories). Proteins were transferred to a polyvinylidene fluoride membrane, blocked in 5% milk in Tris-buffered saline Tween 20, and evaluated with appropriate antibodies (Table S1).

### Luciferase reporter assay

Cells were cotransfected with NF-κB-TATA luciferase (500 ng) and pRL-Renilla luciferase (50 ng) plasmids. After 24 h, transfection media was removed, and cells were exposed to culture medium as indicated. Luciferase activity was measured on a FlexStation3 (Molecular Devices) using a Dual-Luciferase Assay Kit (Promega).

### PCR analysis

Total RNA was extracted with TRIzol (Invitrogen). RNA (1 μg) was reverse transcribed using the High-Capacity cDNA Reverse Transcription Kit (Applied Biosystems) and subjected to quantitative real-time PCR (QuantStudio 12K Flex Real-Time PCR System, Thermo Fisher Scientific; RRID:SCR_021098) using QuantiTect SYBR Green Master Mix (Qiagen). Primer sequences are listed in Table S2. Mean cycle threshold values were determined for control and experimental samples. Changes in mRNA expression were normalized to GAPDH mRNA expression using the 2^−ΔΔCT^ method.

### Statistical analysis

Data are expressed as mean ± SD. Statistical analysis of data with more than two groups were analyzed with two-way ANOVA, and pairwise comparisons were made using the Tukey's test for multiple comparisons. Difference between two groups was determined by unpaired Student’s *t* test. Significance was defined as *p* < 0.05 for all analyses. Specific *p*-values for experimental groups with different means are in Table S3.

## Data availability

All data for this publication are included in the article or are available from the corresponding author upon request.

## Supporting information

This article contains supporting information.

## Conflict of interest

M. D. D is guarantor of this work and, as such, had full access to all the data in the study and takes responsibility for the integrity of the data and accuracy of the data analysis. The authors declare that they have no conflicts of interest with the contents of this article.
