# Supplementary material for: Stress response protein REDD1 promotes diabetes-induced retinal inflammation by sustaining canonical NF-κB signaling
Source: J Biol Chem. 2022 Oct 26;298(12):102638. doi: 10.1016/j.jbc.2022.102638 (PMC9694114; doi:10.1016/j.jbc.2022.102638)
Supplement: Supplemental Materials [file mmc1.pdf]

Supplemental information

REDD1 promotes diabetes-induced retinal inflammation by sustaining canonical NF-κB signaling

Sunilkumar et al

Table of Contents:

1. Table S1: Antibody information

2. Table S2: PCR primer sequences

3. Table S3: Significant differences detected between experimental groups

4. Figure S1: Diabetes induced ICAM-1 and GFAP is attenuated with REDD1 deletion

5. Figure S2: Secondary antibody negative controls for retinal sections

Table S1. Antibody information

Antibody list					
Assay	Antibody	Source	Dilution	Catalog #	Lot#
Western blotting	REDD1	ProteinTech	1:500	10638-1-AP	95508
	ATF4		1:1000	10835-1-AP	00100101
	phospho NF-κB (ser 536)	Cell Signaling	1:1000	3033	17
	NF-κB		1:1000	8242	8
	phospho I-κBα (ser 32)		1:1000	2859	7
	I-κBα		1:1000	4814	17
	phospho IKK α/β (ser176/180)		1:1000	2697	21
	IKKβ		1:1000	2678	2
	phospho IKKγ (ser 376)		1:1000	2689	3
	IKKγ		1:1000	2685	3
	K63 specific Ubiquitin		1:1000	5621	5
	CCL2/MCP-1		1:1000	2029	2
	HA-tag		1:1000	3724	10
	Actin		1:1000	4970	19
	Lamin B	Santa Cruz	1:1000	sc6216	C1307
	GAPDH		1:2000	sc-47724	H1021
	Goat anti-Rabbit IgG h+l-HRP	Bethyl laboratories	1:10000	A120-101	44
	Goat anti-mouse IgG h+l-HRP		1:10000	A90-116	43
	Rabbit anti-goat IgG h+l-HRP		1:10000	A50-100P	29
IF / IHC	F4/80	Cell Signaling	1:200	30325	3
	Iba1/AIF-1 (Alexa Fluor® 647 Conjugate)		1:100	78060	3
	ICAM-1	Santa Cruz	1:200	sc1511	I1014
	GFAP	Dako	1:1000	Z0334	20053562
	Goat anti-Rabbit Alexafluor 488	Jackson	1:1000	711546152	126798
	Goat anti-Rabbit Alexafluor 647		1:1000	711605152	159933
	Donkey anti-goat Alexafluor 488		1:1000	705546147	122088

Table S2. PCR primer sequences

PCR primer list			
Species	Target	Forward sequence (5' - 3')	Reverse sequence (5' - 3')
Mouse	REDD1	GGGATCGTTTCTCGTCCTCC	ATGAGGAGTCTTCCTCCGGC
	CCL2	CACTCACCTGCTGCTACTCA	GCTTGGTGACAAAAACTACAGC
	CCL5	TGCTGCTTTGCCTACCTCTC	TCCTTCGAGTGACAAACACGA
	ICAM-1	AGCCTCCGGACTTCGATCT	TGTTTGTGCTCTCCTGGGTC
	GAPDH	GGTGGTCTCCTCTGACTTCAACA	GTTGCTGTAGCCAAATTCGTTGT
Human	REDD1	CTCTTCGCCCTCGTCCTTG	TCCAGGTAAGCCGTGTCTTC
	ATF4	GGGACAGATTGGATGTTGGAGA	ACCCAACAGGGCATCCAAGT
	CCL2	CATGAAAGTCTCTGCCGCCC	GGGCATTGATTGCATCTGGCTG
	CCL5	TGCTGCTTTGCCTACATTGC	CATCCTTGACCTGTGGACGA
	ICAM-1	TGACCGTGAATGTGCTCTCC	TTCCTTTTTGGGCCTGTTGT
	HO-1	TGACCCATGACACCAAGGAC	GGGGGCAGAATCTTGCACTTT
	GAPDH	GTTGTCTCCTGCGACTTCA	TGCTGTAGCCGTATTCATTG

Table S3: Significant differences detected between experimental groups

	Groups	Adjusted P Value
Figure 1A	REDD1+/+ Veh vs. REDD1+/+ STZ	<0.0001
Figure 1A	REDD1-/- Veh vs. REDD1-/- STZ	0.0003
Figure 1B	REDD1+/+ Veh vs. REDD1+/+ STZ	0.0138
Figure 1D	REDD1+/+ Veh vs. REDD1+/+ STZ	0.0083
Figure 1D	REDD1+/+ STZ vs. REDD1-/- STZ	0.0178
Figure 1E	REDD1+/+ Veh vs. REDD1+/+ STZ	0.0089
Figure 1E	REDD1+/+ STZ vs. REDD1-/- STZ	0.0026
Figure 1F	REDD1+/+ Veh vs. REDD1+/+ STZ	0.0002
Figure 1F	REDD1+/+ STZ vs. REDD1-/- STZ	0.0006
Figure 1G	REDD1+/+ Veh vs. REDD1+/+ STZ	0.0308
Figure 1G	REDD1+/+ Veh vs. REDD1+/+ STZ	0.0011
Figure 2A	WT LG vs. WT HG	0.0068
Figure 2A	WT HG vs. REDD1 KO HG	0.0007
Figure 2B	WT LG vs. WT HG	0.0155
Figure 2B	WT HG vs. REDD1 KO HG	0.0148
Figure 2C	WT LG vs. WT HG	<0.0001
Figure 2C	WT HG vs. REDD1 KO HG	<0.0001
Figure 2D	WT LG vs. WT HG	0.0007
Figure 2D	WT HG vs. REDD1 KO HG	0.0001
Figure 2E	WT vs. REDD1 KO	0.001
Figure 2E	WT vs. REDD1 KO shScr	0.0003
Figure 2E	REDD1 KO shScr vs. REDD1 KO shNrf2	0.0002
Figure 2F	WT vs. REDD1 KO	<0.0001
Figure 2F	WT vs. REDD1 KO shScr	<0.0001
Figure 2F	WT vs. REDD1 KO shNrf2	<0.0001
Figure 2G	WT vs. REDD1 KO	<0.0001
Figure 2G	WT vs. REDD1 KO shScr	<0.0001
Figure 2G	WT vs. REDD1 KO shNrf2	<0.0001
Figure 2H	WT vs. REDD1 KO	<0.0001
Figure 2H	WT vs. REDD1 KO shScr	<0.0001
Figure 2H	WT vs. REDD1 KO shNrf2	<0.0001
Figure 3B	WT Veh vs. WT TNF	0.0009
Figure 3B	WT TNF vs. REDD1 KO TNF	0.0001
Figure 3C	WT Veh vs. WT TNF	0.0005
Figure 3C	WT TNF vs. REDD1 KO TNF	0.0024
Figure 3D	WT Veh vs. WT TNF	<0.0001
Figure 3D	WT TNF vs. REDD1 KO TNF	0.0015
Figure 3D	REDD1 KO Veh vs. REDD1 KO TNF	0.0186

	Groups	Adjusted P Value
Figure 4A	WT Veh vs. WT TNF	<0.0001
Figure 4A	WT TNF vs. REDD1 KO TNF	<0.0001
Figure 4B	WT Veh vs. WT TNF	0.0009
Figure 4B	WT TNF vs. REDD1 KO TNF	0.0011
Figure 4C	WT Veh vs. WT TNF	0.0002
Figure 4C	WT TNF vs. REDD1 KO TNF	0.0003
Figure 4D	WT Veh vs. WT TNF	0.0005
Figure 4D	WT TNF vs. REDD1 KO TNF	0.0008
Figure 4E	Veh vs. TNF	<0.0001
Figure 4E	TNF vs. TNF + IMD	0.0011
Figure 4F	Veh vs. TNF	<0.0001
Figure 4F	Veh vs. TNF + IMD	<0.0001
Figure 4F	TNF vs. TNF + IMD	0.0045
Figure 4G	Veh vs. TNF	0.0049
Figure 4G	TNF vs. TNF + IMD	0.0319
Figure 4H	Veh vs. TNF	<0.0001
Figure 4H	Veh vs. TNF + IMD	0.0031
Figure 4H	TNF vs. TNF + IMD	0.0001
Figure 5A	WT Veh vs. WT TNF	<0.0001
Figure 5A	WT TNF vs. REDD1 KO TNF	0.0012
Figure 5B	WT Veh vs. WT TNF	0.0002
Figure 5B	WT TNF vs. REDD1 KO TNF	0.0003
Figure 6A	REDD1+/+ Veh vs. REDD1+/+ STZ	<0.0001
Figure 6A	REDD1+/+ STZ vs. REDD1-/- STZ	<0.0001
Figure 6B	REDD1+/+ Veh vs. REDD1+/+ STZ	<0.0001
Figure 6B	REDD1+/+ STZ vs. REDD1-/- STZ	0.0002
Figure 6C	REDD1+/+ Veh vs. REDD1+/+ STZ	0.0184
Figure 6C	REDD1+/+ STZ vs. REDD1-/- STZ	0.0841
Figure 6D	REDD1+/+ Veh vs. REDD1+/+ STZ	0.0131
Figure 6D	REDD1+/+ STZ vs. REDD1-/- STZ	0.0131
Figure 6E	REDD1+/+ Veh vs. REDD1+/+ STZ	0.0192
Figure 6E	REDD1+/+ STZ vs. REDD1-/- STZ	0.0044
Figure 6G	REDD1+/+ Veh vs. REDD1+/+ STZ	0.0015
Figure 6G	REDD1+/+ STZ vs. REDD1-/- STZ	0.023
Figure 7A	Veh vs. STZ	0.0316
Figure 7C	WT vs. shATF4	<0.0001
Figure 7D	WT Veh vs. WT TNF	0.0008
Figure 7D	WT TNF vs shATF4 TNF	0.0031
Figure 7E	WT vs. shATF4	0.0173
Figure 7F	WT vs. shATF4	0.0016
Figure 7G	WT vs. shATF4	0.0031
Figure 7H	WT vs. shATF4	0.0002

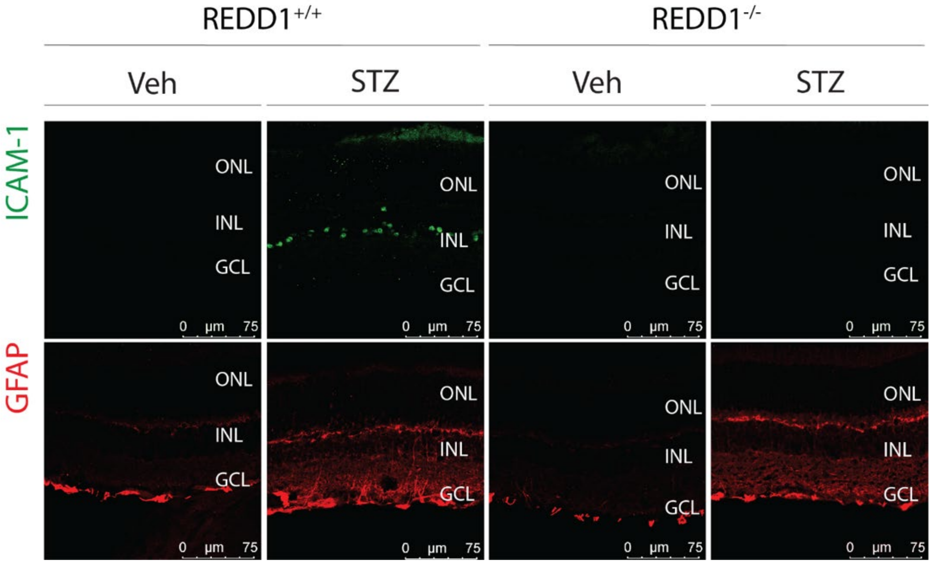

Figure S1. Diabetes induced ICAM-1 and GFAP expression was attenuated by REDD1 deletion. Diabetes was induced in REDD1$^{+/+}$ and REDD1$^{-/-}$ mice by administration of streptozotocin (STZ). All analyses were performed 16 weeks after mice were administered STZ or a vehicle (Veh). Retinas were stained for ICAM-1 (green) and GFAP (red). Representative micrographs are shown (400X magnification; scale bar 75 μm). ONL, outer nuclear layer; INL, inner nuclear layer; GCL, ganglion cell layer.

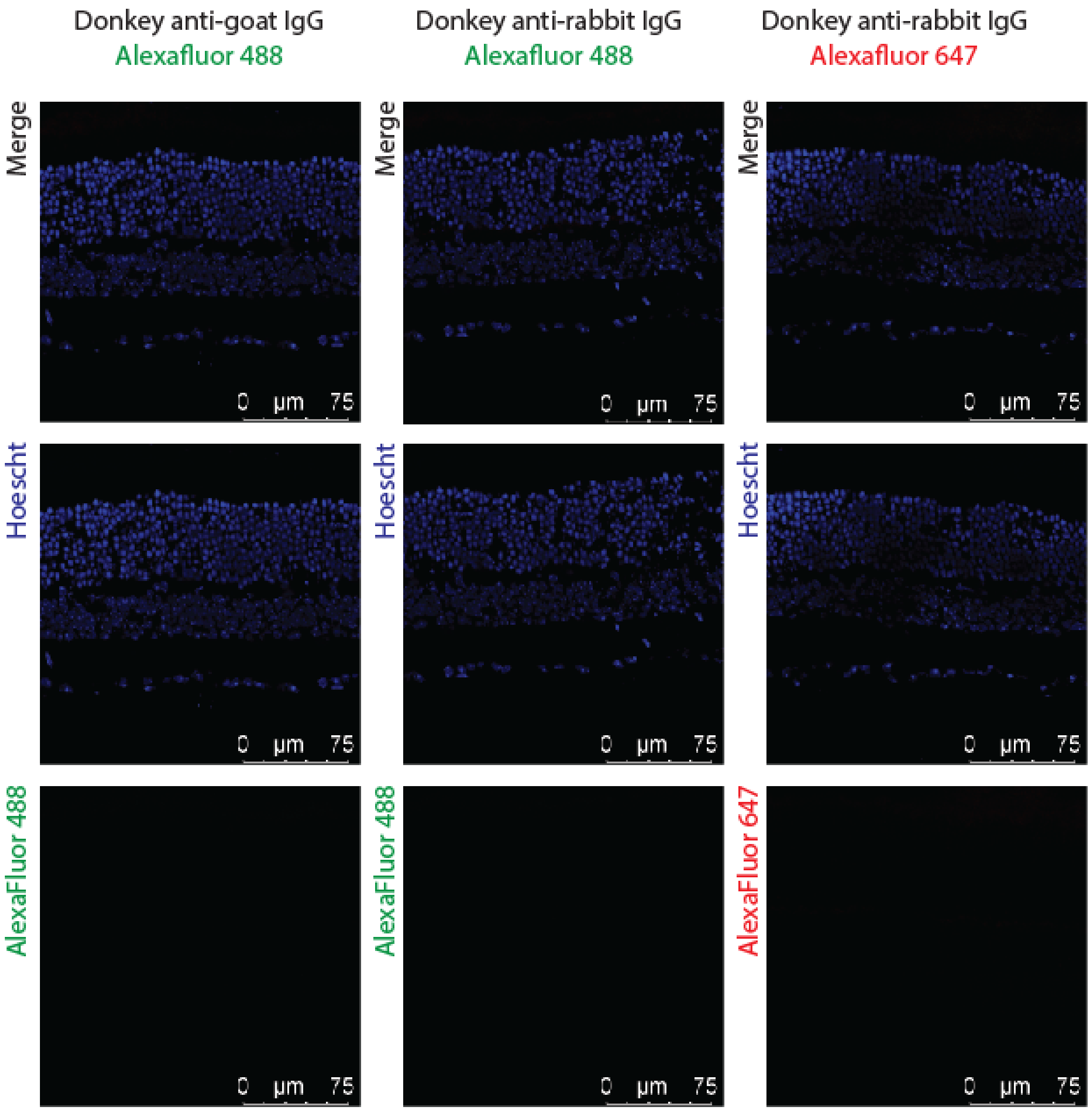

Figure S2. Secondary antibody negative controls for retinal sections. Whole eyes were isolated and sagittally oriented longitudinal cross sections were prepared. Background staining of secondary antibodies was examined in retinal sections by immunofluorescence. Hoechst 33342 (blue) was used to visualize nuclei. Representative micrographs are shown (400X magnification; scale bar 75 μm).
